# Effects of Rehydration on Bacterial Diversity in the Rhizosphere of Broomcorn Millet (*Panicum miliaceum* L.) after Drought Stress at the Flowering Stage

**DOI:** 10.3390/microorganisms12081534

**Published:** 2024-07-26

**Authors:** Yuhan Liu, Jiao Mao, Yuanmeng Xu, Jiangling Ren, Mengyao Wang, Shu Wang, Sichen Liu, Ruiyun Wang, Lun Wang, Liwei Wang, Zhijun Qiao, Xiaoning Cao

**Affiliations:** 1 Center for Agricultural Genetic Resources Research, Shanxi Agricultural University, Taiyuan 030031, China; liuyuhan202305@163.com (Y.L.); maojiao6958@126.com (J.M.); xuyuanmeng1229@163.com (Y.X.); renjiangling0@163.com (J.R.); wangmengyao0113@163.com (M.W.); sj917000@163.com (S.W.); lsch209@163.com (S.L.); 2 College of Agriculture, Shanxi Agricultural University, Jinzhong 030801, China; wry925@126.com (R.W.); wanglun976pzs@sina.com (L.W.); 3 Key Laboratory of Gene Resources and Germplasm Enhancement, Ministry of Agriculture, Taiyuan 030031, China; 4 Institute of Crop Sciences, Chinese Academy of Agricultural Sciences, Beijing 100081, China; wangliwei02@caas.cn

**Keywords:** broomcorn millet, drought stress, rewatering, high-throughput sequencing, rhizosphere, microbial diversity

## Abstract

This study aimed to elucidate responses of the bacterial structure and diversity of the rhizosphere in flowering broomcorn millet after rehydration following drought stress. In this study, the broomcorn millet varieties ‘Hequ red millet’ (A1) and ‘Yanshu No.10′ (A2), known for their different drought tolerance levels, were selected as experimental materials. The plants were subjected to rehydration after drought stress at the flowering stage, while normal watering (A1CK and A2CK) served as the control. Soil samples were collected at 10 days (A11, A21, A1CK1, and A2CK1) and 20 days (A12, A22, A1CK2, and A2CK2) after rehydration. High-throughput sequencing technology was employed to investigate the variations in bacterial community structure, diversity, and metabolic functions in the rhizosphere of the broomcorn millet at different time points following rehydration. The findings indicated that the operational taxonomic units (OTUs) of bacteria in the rhizosphere of broomcorn millet were notably influenced by the duration of treatment, with a significant decrease in OTUs observed after 20 days of rehydration. However, bacterial Alpha diversity was not significantly impacted by rehydration following drought stress. The bacterial community in the rhizosphere of broomcorn millet was mainly composed of *Actinobacteria* and *Proteobacteria*. After rewatering for 10 to 20 days after drought stress, the abundance of *Sphingomonas* and *Aeromicrobium* in the rhizosphere soil of the two varieties of broomcorn millet decreased gradually. Compared with Yanshu No.10, the abundance of *Pseudarthrobacter* in the rhizosphere of Hequ red millet gradually increased. A Beta diversity analysis revealed variations in the dissimilarities of the bacterial community which corresponded to different rehydration durations. The relative abundance of bacterial metabolic functions in the rhizosphere of broomcorn millet was lower after 20 days of rehydration, compared to measurements after 10 days of rehydration. This observation might be attributed to the exchange of materials between broomcorn millet and microorganisms during the initial rehydration stage to repair the effects of drought, as well as to the enrichment of numerous microorganisms to sustain the stability of the community structure. This study helps to comprehend the alterations to the bacterial structure and diversity in the rhizosphere of broomcorn millet following drought stress and rehydration. It sheds light on the growth status of broomcorn millet and its rhizosphere microorganisms under real environmental influences, thereby enhancing research on the drought tolerance mechanisms of broomcorn millet.

## 1. Introduction

Drought is one of the abiotic factors that limit plant growth and yield [1]. In recent years, crop production has been increasingly restricted by water resources [2,3]. Improving the survival ability of crops under drought stress is essential for the growth and survival of crop species [4,5]. The rhizosphere is important for plants in terms of absorbing water and nutrients, and rhizosphere microorganisms, as an important part of the rhizosphere, significantly affect the interaction between plants and the soil environment [6,7]. When drought stress occurs, rhizosphere microorganisms can regulate plant growth, promote photosynthesis [8], and enhance the drought tolerance of host plants [9]. Cantabella found that microorganisms can stimulate root growth by triggering root development signals [10] and assist plants in improving the absorption of water and nutrients through the roots so as to better adapt to adversity stress. PGPR strains can promote plant growth under drought conditions by reducing ACC and ethylene levels in plants [11]. Arbuscular mycorrhizal fungi (AMF) can improve the oxidative damage induced by drought in maize [12], affect the hormone secretion of plants, and enhance the drought resistance of plants [13].

Broomcorn millet (*Panicum miliaceum* L.) is an important food crop in arid and semiarid areas [14]. Compared with other crops, broomcorn millet has a stronger tolerance to drought [14]. When drought occurs, broomcorn millet will produce a series of physiological and biochemical reactions to resist the threat of drought [15]. The expression levels of energy metabolism, anthocyanin, and photosynthesis, as well as plant-hormone-related genes closely related to the drought resistance of broomcorn millet, are increased. These substances can adapt to drought by accurately regulating various molecular pathways [16,17]. As an ideal crop for studying the mechanisms involved in drought tolerance [18], the composition of its rhizosphere’s microbial community [19] and various influencing factors have been studied. Tiao‘s research showed that annual average temperature and soil pH were important driving factors in the regulation of the rhizosphere community, key species, and modularization of broomcorn millet [20]. Soil nutrients [21], planting methods [22], and growth stages of broomcorn millet [23] also affected the microbial diversity and community structure of the rhizosphere.

In actual production, plants usually grow in alternating wet and dry environments [24,25]. The rainfall in the main producing areas of broomcorn millet has a certain seasonality [26] and is mostly concentrated in the heading and flowering period of broomcorn millet, which is also the period when broomcorn millet needs the most water. Therefore, it is of great importance to explore the physiological response mechanisms of broomcorn millet under drought stress and rehydration conditions to reveal the physiological changes in broomcorn millet under natural conditions. Zhao found that broomcorn millet could quickly repair root cap function and increase net photosynthetic rate [27] to repair the effects of drought stress after rewatering subsequent to drought stress at the jointing stage [28]. Although this is a key period for millet growth, few studies have focused on the physiological, molecular, and other mechanisms present in millet after drought and rehydration during heading and flowering. The flowering period is also the most abundant period in terms of rhizosphere microorganisms [29,30]; however, research on rhizosphere microorganisms under drought stress during the flowering stage has mainly focused on the changes in the microbial population diversity of the rhizosphere [31] and the prediction of metabolic function [29]. In sum, research related to the effect of rewatering on the rhizosphere microorganisms of broomcorn millet after drought stress during the flowering stage is still undeveloped. In this study, two kinds of broomcorn millet with different drought tolerance levels were rewatered after drought stress during the flowering stage. The diversity, structure, and metabolic function of rhizosphere microorganisms of broomcorn millet at 10 days and 20 days after rewatering were analyzed to explore the effects of rewatering after drought on the diversity and structure of rhizosphere microorganisms of different varieties. It is helpful to understand the change trends in rhizosphere microorganisms in broomcorn millet after rewatering, a problem which is of great theoretical significance in terms of improving research on the drought tolerance mechanisms of broomcorn millet and the theoretical aspects of organic dry farming.

## 2. Materials and Methods

### 2.1. Test Materials, Drought Stress Treatment and Sample Collection

The experiment was carried out at the Hequ red millet Experimental Base of the Agricultural Gene Resources Research Center of Shanxi Agricultural University (39°080′20.78″ N, 110°14′018.74″ E). The drought-tolerant variety Hequ red millet (A1) and the drought-intolerant variety Yanshu No.10 (A2) were used as the experimental materials [29,30], and 10 kg of air-dried soil was loaded per pot. Soil samples (sandy loam) were collected from local farmland (pH 8.41; organic matter, 8.17 g/kg; total nitrogen, 0.69 g/kg; available phosphorus, 5.87 mg/kg; available potassium, 97.07 mg/kg; alkali-hydrolyzable nitrogen, 53.6 mg/kg). The collection area was about 20–30 m^2^, the collection depth was about 20 cm, the diameter was 5 cm, the length was about 20 cm, and a soil drill was used for collection, This plot had not been used to plant broomcorn millet before. The field soil was dried and sieved to 2 mm to remove rock and plant debris.

Before sowing, broomcorn millet seeds were disinfected, bleached for 5 min, and washed with sterile water at least 3 times. All of the experiments were carried out in a dry shed. Five robust broomcorn millet plants were retained in each pot. The experiment adopted a completely randomized block design. The drought-treated pots were subject to controlled water allocations during the broomcorn millet heading stage. The drought treatment lasted for 4 days in order to reach the level of severe drought stress (soil water content of 15% [30]). Subsequently, the weight was measured every two days, and the soil water content for the broomcorn millet was thereby maintained at 15%. The control treatment was weighed every two days to ensure that the water content was 55%. Irrigation was provided using sterile water throughout the process.

After 15 days of drought stress, the drought-treated pots were watered, and soil samples were taken after 10 days and 20 days, respectively. After 10 days of rewatering after drought stress, the samples taken were denoted A11 and A21, and the control treatments were denoted A1CK1 and A2CK1. The samples taken after 20 days of rewatering after drought stress were denoted A21 and A22, and the control treatments were denoted A1CK2 and A2CK2. Each of the 5 pots was resampled once, and each treatment was repeated three times. Loose soil was removed when sampling, and roots were flushed with 5 mL of 0.9% NaCl solution. The resulting solution was centrifuged at 4 °C and 12,000 rpm for 10 min, and the sediment was defined as a rhizosphere soil sample. These rhizosphere soil samples were then transferred to a 5 mL sterile tube and stored at −20 °C pending further analysis.

### 2.2. DNA Extraction, PCR Amplification, and Illumina MiSeq Sequencing

The E-Z 96^®^ Mag-Bind Soil DNA Kit (Omega Bio-Tek, Inc., Guangzhou, China) was used to extract DNA and perform PCR amplification. The primers were ‘338F: ACTCCTACGGGAGGCAGCA’ and ‘806R: GGACTACHVGGGTWTCTAAT’. The sequencing was completed by Shanghai Pasenuo Biotechnology Co., Ltd. (Shanghai, China). The Q5 high-fidelity DNA polymerase from NEB (NewEnglandBiolabs, Inc., Ipswich, MA, USA) was used for PCR amplification, and the number of amplification cycles was strictly controlled to make the number of cycles as low as possible, while ensuring that the amplification conditions of the same batch of samples were consistent.

### 2.3. Original Data Processing, Operation Classification Unit Division, and Diversity Analysis

The obtained sequencing data were identified and spliced using QIIME 1.8.0 software (Quantitative Insights into Microbial Ecology), and low-quality, non-specific amplified sequences and chimeric sequences were removed [31]. The UCLUST sequence alignment tool [32] was used to merge and divide the obtained sequences according to 97% sequence similarity, and the sequence with the highest abundance in each OTU was selected as the representative sequence of the OTU. Then, according to the number of sequences contained in each OTU in each sample, a matrix file [OTU table] of OTU abundance in each sample was constructed. A species annotation analysis of OTUs was then performed based on the UNITE database [32] to obtain the taxonomic information of each OTU and construct the dilution curve, species accumulation curve, and grade abundance curve. The α diversity index was calculated using QIIME software, including the richness index Chao1 [33], the Shannon diversity index, the Simpson index, and the ACE index [33].

Beta diversity analysis was used to test the similarity of the community structures between different samples, mainly through the use of principal component analysis (PCA) [34].

### 2.4. Prediction of the Metabolic Function of Microbial Community

The PICRUSt method was used to compare the existing 16S rRNA gene prediction data with the microbial reference gene database with known metabolic functions. This was carried out to predict the metabolic function of the bacteria. Based on the full-length 16S rRNA gene sequence of the microorganisms, the gene function profiles of common ancestors with significantly differentially expressed OTUs were predicted [35]. The functional spectra of other relevant untested species in the full-length sequence database of Greengenes were inferred for the 16S rRNA gene, and the functional spectrum of the bacterium was constructed. Then, the 16S rRNA gene sequence data obtained via sequencing were compared with the Greengenes database to find the nearest neighbor of the reference sequence of each sequence and classify this neighbor as a reference OTU. The OTU abundance matrix was corrected according to the rRNA gene copy number of the nearest neighbor of the reference sequence, and the bacterial composition data were mapped to a database of known gene function profiles to predict the metabolic function of the bacterium.

### 2.5. Statistical Analysis

One-way analysis of variance was used to test whether there were significant differences in the data between different treatments (*n* = 3). IBM SPSS Statistics (version 20.0) (SPSS 2011) was used for multivariate analysis of variance. The individual effects and interaction-based effects of different treatments, varieties, and sampling periods on the OTUs and the Alpha diversity of broomcorn millet rhizosphere bacteria were investigated. A one-way analysis of variance was used to compare the groups. The data were expressed as mean ± standard deviation (SD), and *p* < 0.05 was considered significant.

## 3. Results

### 3.1. Effects of Rewatering after Drought Stress on Bacterial Community Diversity in the Rhizosphere of Broomcorn Millet

The OTUs of each treatment ranged from 7937 ± 328.28 to 9111 ± 673.66 (mean ± SD), and the Shannon index ranged from 8.83 ± 0.19 to 9.13 ± 0.09. The sampling period after rehydration had a significant effect on the OTUs of rhizosphere bacteria in broomcorn millet, while the differences in broomcorn millet varieties and treatments had no significant effects on the OTUs of the rhizosphere bacteria. There were no significant differences in the Alpha diversity of broomcorn millet rhizosphere bacteria from the influence of differences in broomcorn millet varieties, treatments, and sampling periods, and the interaction between the three had no significant effect on the OTUs and Alpha diversity of broomcorn millet rhizosphere bacteria (Table 1).

### 3.2. Changes in Bacterial Abundance

**Figure 1:**
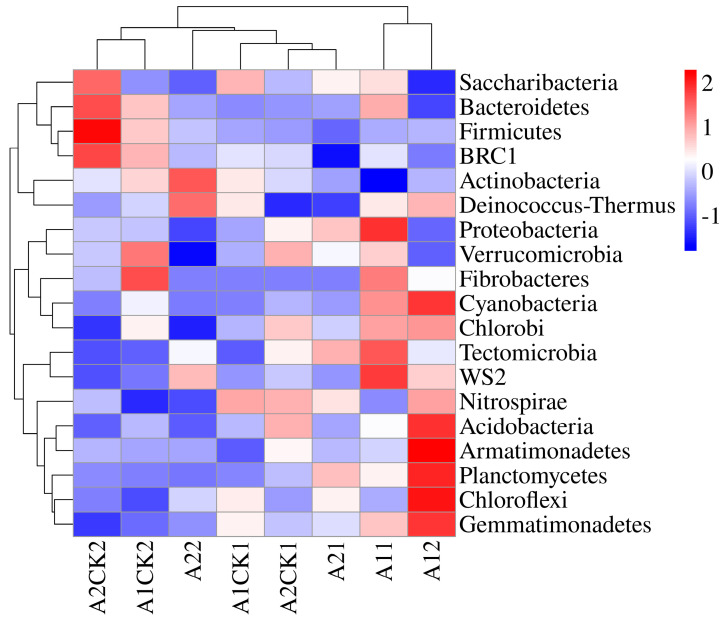
Heatmap of horizontal clustering of rhizobacteria. A12, A22: rewatering for 20 days after drought stress; A1CK1, A2CK1: no stress, rehydration for 10 days; A1CK2, A2CK2: rewatering for 20 days, and without stress.

Based on the OTU classification results, the rhizosphere bacteria of broomcorn millet mainly comprised *Actinobacteria*, *Proteobacteria*, *Chloroflexi*, *Gemmatimonadetes*, *Acidobacteria*, *Bacteroidetes*, *Firmicutes*, *Saccharibacteria*, *Verrucomicrobia*, and *Planctomycetes*. In all samples, these bacterial phyla were the 10 most dominant phyla detected in this study, accounting for 98.87–99.44% of the total. By analyzing the changes in the relative abundance of microorganisms, differences in microbial enrichment groups under two different growth conditions were found (Figure 1). Drought stress had no significant effect on the main bacteria in the rhizosphere of broomcorn millet, but the abundance of some bacteria fluctuated.

At the phylum level, the abundance of *Actinobacteria* and *Firmicutes* in the rhizosphere soil of broomcorn millet increased after rehydration treatment, while the abundance of *Proteobacteria*, *Saccharibacteria*, and *Verrucomicrobia* decreased (Figure 1). Compared with Yanshu No.10, the abundance of *Chloroflexi*, *Gemmatimonadetes*, and *Acidobacteria* in the rhizosphere soil of Hequ red millet increased gradually after rehydration, while the abundance of *Bacteroidetes* decreased gradually. The abundance of *Bacteroidetes* in the rhizosphere bacteria of Yanshu No.10 increased gradually after rehydration, and the abundance of *Chloroflexi*, *Gemmatimonadetes,* and *Acidobacteria* decreased gradually.

**Figure 2:**
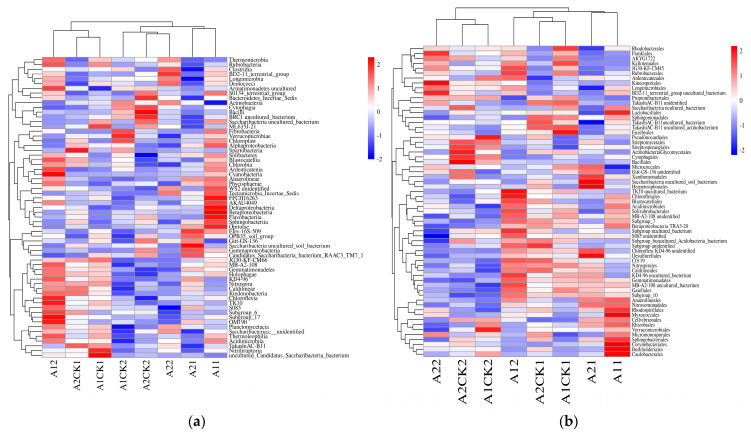

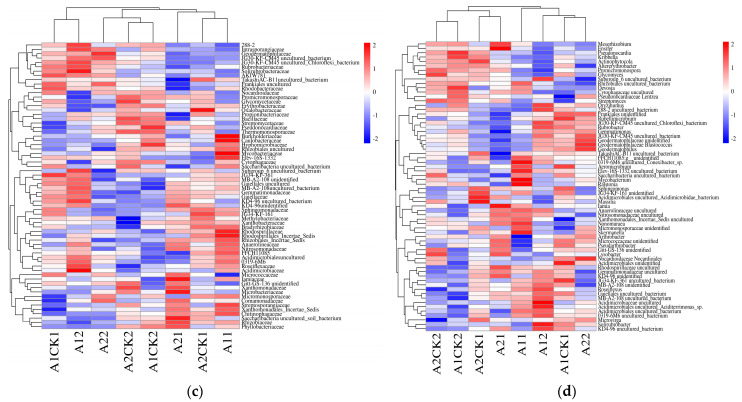
The first 50 cluster heatmaps of bacterial abundance in the rhizosphere of broomcorn millet. (**a**): class-level clustering heatmap of rhizosphere bacteria; (**b**): cluster heat map of rhizosphere bacteria at the order level; (**c**): family-level clustering heatmap of rhizosphere bacteria; (**d**): cluster heatmap of rhizosphere bacteria at the genus level. A11, A21: rewatering for 10 days after drought stress; A12, A22: rewatering for 20 days after drought stress; A1CK1, A2CK1: no stress, rehydration for 10 days; A1CK2, A2CK2: rewatering for 20 days, and without stress.

At the level of class classification, most of the bacteria in the rhizosphere soil of broomcorn millet were *Actinobacteria*, *Alphaproteobacteria*, and *Thermoleophilia*. After rehydration treatment, the abundance of *Actinobacteria*, *Thermoleophilia*, and *Acidimicrobiia* in the rhizosphere soil of broomcorn millet increased, while the abundance of *Alphaproteobacteria*, *Gammaproteobacteria*, and *Betaproteobacteria* decreased gradually (Figure 2a). Compared with Yanshu No.10, the abundance of *Gemmatimonadetes*, KD4-96, and Gitt-GS-136 in the rhizosphere soil of Hequ red millet increased gradually after rehydration, and the abundance of *Cytophagia*, *Deltaproteobacteria*, and Takashi AC-B11 decreased gradually. The abundance of *Cytophagia*, *Deltaproteobacteria*, and Takashi AC-B11 in the rhizosphere bacteria of Yanshu No.10 increased gradually after rehydration, and the abundance of *Gemmatimonadetes*, KD4-96, and Gitt-GS-136 first increased and then decreased.

At the order level, the bacteria in the rhizosphere soil of broomcorn millet were mainly composed of *Micrococcales*, *Rhizobiales*, *Acidimicrobiia*, *Propionibacteriales*, and *Frankiales*. After the rewatering treatment, the abundance of *Acidimicrobiales*, *Frankiales,* and *Solirubrobacterales* in the rhizosphere soil of broomcorn millet increased gradually, while the abundance of *Rhizobiales* and *Sphingomonadales* decreased gradually (Figure 2b). Compared with Yanshu No.10, the abundance of *Micrococcales* and *Gemmatimonadales* in the rhizosphere soil of Hequ red millet increased gradually after rehydration. The abundance of *Propionibacteriales* and *Pseudonocardiales* decreased gradually. The abundance of *Propionibacteriales* and *Pseudonocardiales* in the rhizosphere bacteria of Yanshu No.10 increased gradually after rehydration, while the abundance of *Micrococcales* and *Gemmatimonadales* decreased gradually.

At the family level, the bacteria in the rhizosphere soil of broomcorn millet were mainly composed of *Micrococcaceae*, *Nocardioidaceae*, *Geodermatophilaceae*, *Pseudonocardiaceae*, and *Sphingomonadaceae*. After the rewatering treatment, the abundance of *Geodermatophilaceae*, *Solirubrobacteraceae*, and *Methylobacteriaceae* in the rhizosphere soil of broomcorn millet increased gradually, while the abundance of *Sphingomonadaceae*, *Xanthomonadaceae*, and JG34-KF-161 decreased gradually (Figure 2c). Compared with Yanshu No.10, the abundance of *Micrococcaceae*, *Gemmatimonadaceae*, and *Streptomycetaceae* in the rhizosphere soil of Hequ red millet after the rewatering treatment increased gradually, and the abundance of *Nocardioidaceae* and *Pseudonocardiaceae* decreased gradually. The abundance of *Nocardioidaceae* and *Pseudonocardiaceae* in the rhizosphere bacteria of Yanshu No.10 increased gradually after rehydration, and the abundance of *Micrococcaceae* and *Gemmatimonadaceae* decreased gradually.

At the genus level, the bacteria in the rhizosphere soil of broomcorn millet were mainnly composed of *Blastococcus*, *Nocardioides*, *Sphingomonas*, and *Pseudarthrobacter*. After rehydration treatment, the abundance of *Blastococcus* and *Nocardioides* in the rhizosphere soil of broomcorn millet increased gradually, while the abundance of *Sphingomonas*, *Aeromicrobium*, and *Lysobacter* decreased gradually (Figure 2d). Compared with Yanshu No.10, the abundance of *Pseudarthrobacter*, *Streptomyces*, and *Roseiflexus* in the rhizosphere soil of Hequ red millet increased gradually after rehydration. The abundance of *Pseudonocardia* and *Mycobacterium* in the rhizosphere bacteria of Yanshu No.10 increased gradually after rehydration, while the abundance of *Pseudarthrobacter*, *Streptomyces*, and *Roseiflexus* decreased gradually.

**Figure 3:**
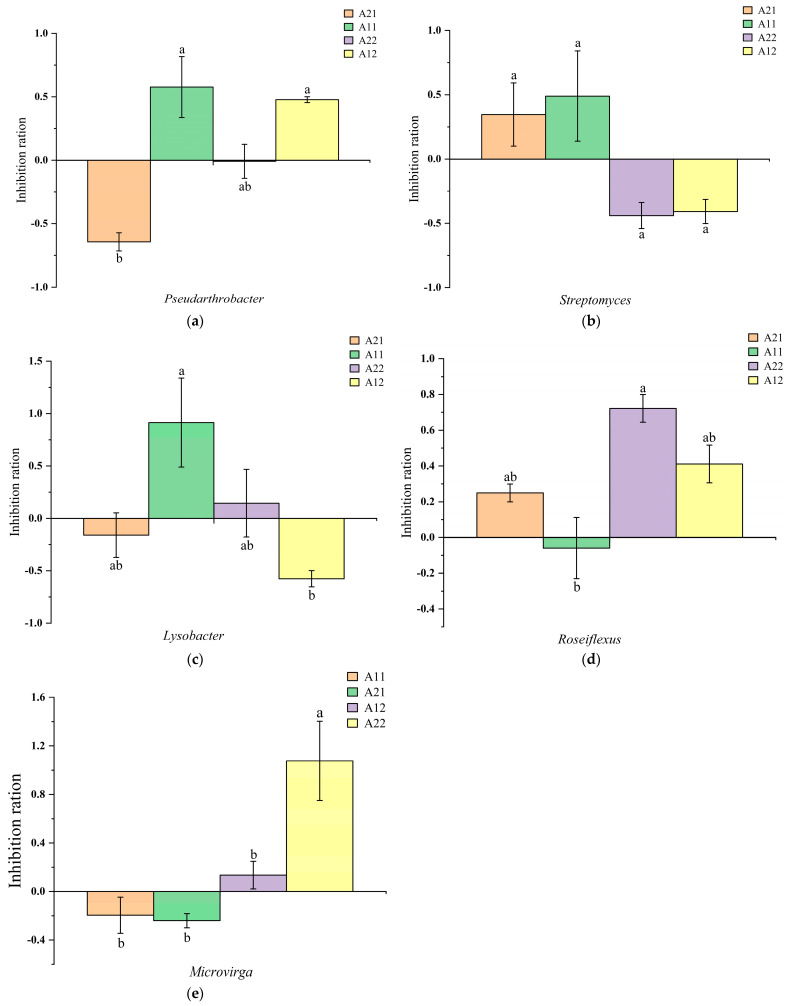
The effect of rewatering after drought stress on the relative abundance of bacteria in the rhizosphere of broomcorn millet at the genus level. The formula for calculating the multiple change is as follows: (relative abundance under drought stress rewatering conditions/relative abundance under control conditions)-1. The error bars represent three independently repeated standard errors. Different lowercase letters indicated that the expression level was significantly different at the *p* < 0.05 level. (**a**): *Pseudarthrobacter*; (**b**): *Streptomyces*; (**c**): *Lysobacter*; (**d**): *Roseiflexus*; (**e**): *Microvirga*.

Rewatering for 10 and 20 days after drought stress had different effects on the relative abundance of special bacteria in the rhizosphere of broomcorn millet (Figure 3). After 10 days of rehydration, the abundance of *Pseudarthrobacter* in the rhizosphere of Hequ red millet was significantly inhibited. After 20 days of rehydration, the abundance of *Pseudarthrobacter* was significantly inhibited by the external environment (Figure 3a). The relative abundance of this genus gradually increased after rewatering of broomcorn millet under drought stress. The bacteria in the rhizosphere soil of Yanshu No.10 were not significantly inhibited, but the inhibition was enhanced after 20 days of rewatering, and the relative abundance decreased after rewatering under drought stress. The relative abundance of *Streptomyces* varied with the intensity of inhibition after rewatering for 10 days and 20 days (Figure 3b). The relative abundance of *Lysobacter* bacteria in the rhizosphere of Hequ red millet was inhibited after 10 days of rehydration, and the relative abundance then gradually decreased. The relative abundance of *Lysobacter* bacteria in Yanshu No.10 soil gradually decreased (Figure 3c). The relative abundance of *Roseiflexus* bacteria in the rhizosphere soil of Hequ red millet gradually increased (Figure 3d), and the relative abundance of *Microvirga* bacteria in the rhizosphere soil of broomcorn millet gradually increased (Figure 3e).

**Figure 4:**
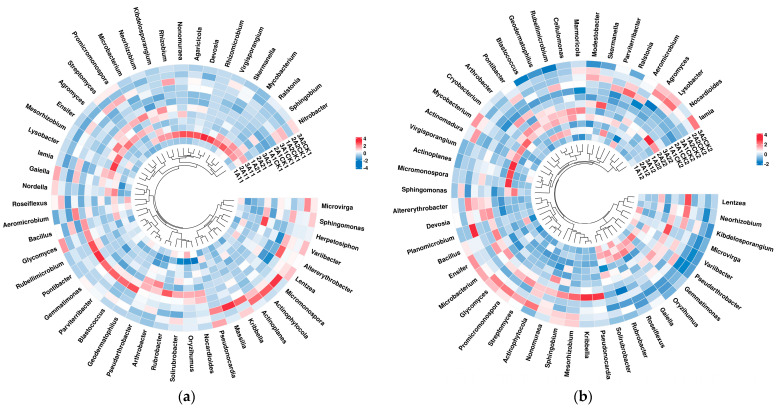
Classification analysis of the genus heatmap of the top 50 levels of abundance. (**a**,**b**): Heat map and cluster analysis of the comprehensive population composed of samples taken 10 days and 20 days after rehydration, respectively.

The main groups of bacteria in the rhizosphere of broomcorn millet did not change after rehydration. The most abundant phyla in the rhizosphere soil of broomcorn millet were *Actinobacteria* and *Proteobacteria*, and the most abundant class was *Actinobacteria*. Cluster analysis of the top 50 genera in different samples was carried out according to the abundance distribution of taxa or the similarity between samples. It was found that the top 50 genera of rhizosphere bacteria exhibited a dynamic change trend after rewatering for 10 days and 20 days. After 10 days of rewatering, the genera with high abundance in the rhizosphere bacteria of Hequ red millet were *Neorhizobiu*, *Kibdelosporangium*, *Rhizobium*, and so on. The most abundant bacteria in the rhizosphere of Yanshu No.10 were *Lysobacter* and *Herpetosiphon*. After 20 days of rehydration, the genus with the highest abundance in the rhizosphere of Hequ red millet became *Gemmatimonas*, while the genus with highest abundance in the rhizosphere of Yanshu No.10 was *Micromonospora* (Figure 4a,b).

### 3.3. Changes in Beta Diversity of Rhizosphere Bacteria in Broomcorn Millet after Rewatering under Drought Stress

**Figure 5:**
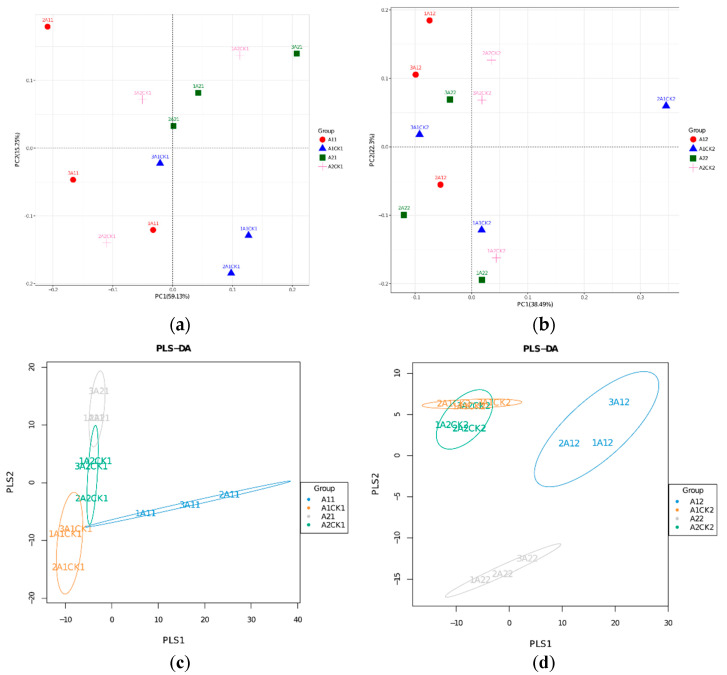
Analysis of soil microbial Beta diversity. (**a**,**b**): Two-dimensional ordination of principal component analysis of rhizosphere microorganisms after 10 days and 20 days of rewatering; (**c**,**d**): PLS-discriminant analysis of graded microorganisms after 10 days and 20 days of rehydration. Identical groups of samples are marked with ellipses.

In order to observe the dissimilarity of bacterial communities between different samples, the Beta diversity of samples was analyzed via principal component analysis (PCA). It was found that the cumulative contribution rates of principal component variance in the two periods reached 74.38% and 60.79%. The cumulative variance contribution rate of the two principal components is less than 80%. Since the method is based on use of the covariance matrix to find the correlation, it can still be used to explain a relationship that explains a variance less than 80%. Further analysis showed that the microbial community structure of A21 and A1CK1 rhizosphere samples was relatively similar at 10 days after rewatering (Figure 5a), while the results for the A22 and A2CK2 rhizosphere microbial communities were similar at 20 days after rewatering (Figure 5b). A large number of microbial community data generated via high-throughput sequencing based on species abundance matrix and sample grouping data were analyzed using partial least squares discrimination analysis (PLS-DA). The results showed that the rhizosphere microbial communities of A21 and A2CK1 were more similar to each other after 10 days of rewatering (Figure 5c), while A1CK2 and A2CK2 were more similar to each other after 20 days of rewatering (Figure 5d).

### 3.4. Effects of Rewatering after Drought Stress on the Metabolic Function of Rhizosphere Bacteria

The high-throughput sequencing information was compared with the KEGG (KEGG Pathway Database, http://www.genome.jp/kegg/pathway.html, accessed on 24 July 2024) database (Kyoto Encyclopedia of Genes and Genomes), and PICRUSt was used to predict the functions of the bacterial communities in the four soils. The results were annotated to six primary functional metabolic pathways, namely, Metabolism, Genetic Information Processing, Environmental Information Processing, Cellular Processes, Organismal Systems and Human Diseases. There are 41 secondary functional metabolic pathways. The third level corresponds to the metabolic pathway map, while the fourth level corresponds to the specific annotation information of each KO (KEGG orthologous groups, KEGG orthologous gene clusters) on the metabolic pathway.

**Figure 6:**
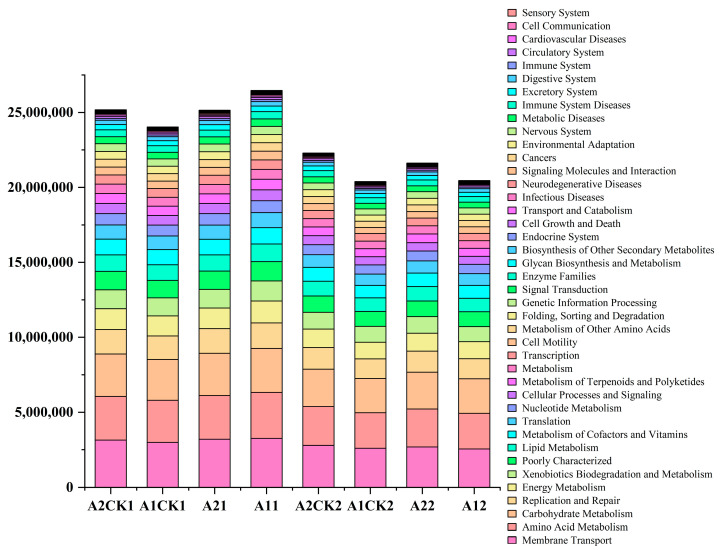
Difference analysis of rhizosphere bacterial metabolic pathways.

The main secondary metabolic functions of broomcorn millet rhizosphere bacteria include the following: Membrane Transport, Amino Acid Metabolism, Carbohydrate Metabolism, Replication and Repair, etc. (Figure 6). After rewatering, there was no significant change in the types of rhizosphere microbial metabolic functions. The relative abundance of cell communication increased in the metabolic pathway of Yanshu 10 rhizosphere bacteria after rewatering after drought stress. The relative abundance of other metabolic functions decreased.

## 4. Discussion

The response mechanism of plants to water stress involves the expression of many functional and regulatory genes [36], decreased physiological photosynthetic activity, increases in transcriptional activator MYC and MYB proteins [37], and the accumulation of fructan [38] and other substances in the plants. The structure and diversity of rhizosphere microorganisms in plants will also change accordingly [39]. On the one hand, it maintains its own growth and ecological network [40]. On the other hand, it strengthens the interaction with plants and carries out post-drought restoration work. When the external environment changes, a variety of drought traits will change in plants, from root and leaf traits to osmotic adjustment ability, water potential, plant hormone content, etc. [41]. These traits interact with the associated rhizosphere microorganisms through flavonoids, comarins, N-containing compounds, and terpenes secreted by the plants [42,43]. Therefore, it is very important to explore the interaction between plants and microorganisms and their response to rehydration after drought stress.

Based on high-throughput sequencing technology, this study analyzed the differences in the rhizosphere of broomcorn millet after 10 days and 20 days of rewatering after drought stress treatment. The results showed that there was no significant difference in bacterial diversity in the rhizosphere of broomcorn millet under the different treatments after 10 days of rewatering (Table 1), which was consistent with the results of previous studies on drought and rewatering of *Hibiscus rosa-sinensis* [44]. We believe that there are three reasons for this result. First, broomcorn millet may directly recruit beneficial microorganisms in the rhizosphere after 10 days of rehydration to repair the losses caused by stress. Plants can secrete special substances to affect microbial communities [45] and selectively enrich them [46]. For example, legumes are symbiotic with rhizobia via the secretion of flavonoids under nitrogen stress [47]; providing carbon sources to the rhizosphere can enable arbuscular mycorrhizal fungi to occupy the growth sites of pathogenic microorganisms [42]. At the same time, rhizosphere microorganisms can also directly respond to stress and secrete volatile organic compounds to help plants resist stress threats [48]. Secondly, the community diversity detected under the condition of rewatering after drought stress may represent the normal state of growth and the normal rhizosphere microorganisms of broomcorn millet in arid and semiarid areas [49,50]. In the face of drought stress, the internal starch, protein, thiamine, and nicotinamide, as well as other physiological indicators of broomcorn millet, changed [16]. These indicators may affect the distribution of carbon assimilation in the underground parts of broomcorn millet and indirectly affect the diversity of rhizosphere microorganisms [51], a process which, in turn, affects the rhizosphere environment and broomcorn millet itself. Chemical signals released by host plants can stimulate the rapid germination of microorganisms and establish beneficial interactions with them [52,53]. Harrison‘s study showed that vesicular–arbuscular (VA) can form a symbiotic relationship with terrestrial plants and help host plants absorb Pi [54]. Some plants, such as legumes, and rhizobia [55], use signal substances between them to achieve plant microorganism synergistic symbiosis. There is also signal transduction between PGPR and plants, allowing PGPR to interact with plants and help plants improve their tolerance [56]. Therefore, we speculate that there is a certain signal transduction between broomcorn millet and its local microbial population, which may be due to the co-evolution of the two over time [30]. Third, short-term drought stress treatment did not affect broomcorn millet and its rhizosphere environment, and subsequently, the bacterial diversity in the rhizosphere of broomcorn millet did not show a significant difference after rehydration [29,30]. Therefore, the specific fluctuation of broomcorn millet bacteria during drought and rewatering and the specific physiological mechanism of material exchange between the two are still unclear. It is necessary to sample many times in the time after rewatering to understand the changes and causes relevant to bacteria.

There were differences in the abundance and variation of rhizosphere bacteria during the rehydration process of broomcorn millet. The results of the taxonomic analysis showed that the number of OTUs in rhizosphere bacteria after 20 days of rewatering was slightly lower than that of the sample taken after 10 days, and the relative abundance levels of some bacteria were different (Figure 1 and Figure 2). After 20 days of rehydration, the abundance of *Microvirga* bacteria in the broomcorn millet soil gradually increased, and the abundance of *Lysobacter* and *Sphingomonas* gradually decreased (Figure 2 and Figure 3). Maghboli studies have shown that *Microvirga* bacteria can improve plant rhizome growth [57]. There are some genes involved in a plant growth-promoting [PEP] mechanism in the genome of *Microvirga brassicacearum* CDVBN77^T^. The genome annotation shows the gene encoding phosphatase active protein [58], which promotes the absorption of nutrients by plants and promotes plant growth. *Lysobacter* genus bacteria can use secreted antibiotics and growth inhibitory enzymes to prey on other harmful bacteria [59]. *Lysobacter enzymogenes* strain C3 can produce a variety of extracellular β-1,3-glucanases encoded by gluA, gluB, and gluC genes to degrade the cell walls of filamentous fungi [60,61], thereby killing pathogenic bacteria. Long‘s study showed that *Lysobacter enzymogenes* OH11 can secrete heat-resistant antifungal factor [HSAF] [62] to control a variety of plant Phytophthora diseases caused by oomycetes, such as *Phytophthora sojae* [63], promote plant resistance to a variety of Phytophthora pathogens, and reduce the impact of pathogens on plant growth and yield.

The *Sphingomonas* genus bacteria can increase antioxidant enzyme activity and osmotic adjustment substances. Studies conducted by Finkel have shown that *Sphingomonas* sp. LK11 can encode tryptophan synthesis gene clusters (trpA, trpB, and trpD) and indolylpyruvate ferredoxin oxidoreductase (IOR; they are site AV944_07715 and site AV944_07710) and other related genes that promote plant growth [64]. Trehalose biosynthesis pathways [otsA/otsB and treY/treZ] exist in its genome, and trehalose acts as an osmoprotectant to protect plants under various stresses [65,66]. There are also many salt-tolerant genes that can protect plant cells by encoding the betT choline transporter, β-choline dehydrogenase, betB betaine aldehyde dehydrogenase, and choline synthetic osmolyte betaine [67,68]. In the rhizosphere environment, *Sphingomonas* sp. Hbc-6 bacteria can promote plant growth, increase the richness and diversity of plant rhizosphere bacterial communities, and make the community more complex to resist stress [69]. At the same time, *Sphingomonas* sp. Hbc-6 can recruit more bacteria which are potentially beneficial to the rhizosphere. For example, *Variovorax* can regulate plant hormone levels to balance the effects of ecologically realistic synthetic root communities on root growth [70]. *Pseudomonas* promotes the absorption of Pi by plants [71]. *Methylobacterium* produces plant hormones to promote plant proliferation, affect seed germination, help plants resist water stress [72,73], and jointly promote plant growth under different conditions. *Sphingomonas* sp. Cra20 can selectively increase the growth rate of plants, increase the growth of lateral roots and root hairs of plant roots [74], produce a certain enrichment effect on microorganisms, and improve bacterial diversity, and thus change the rhizosphere bacterial community. Therefore, the rhizosphere microorganisms of broomcorn millet may improve the growth and drought tolerance of broomcorn millet by changing its richness and diversity.

In the rhizosphere microorganisms of different genotypes of broomcorn millet, there were different changes in the abundance levels of different bacteria (Figure 3). The abundance of *Pseudarthrobacter*, *Streptomyces,* and *Roseiflexus* in the rhizosphere soil of Hequ red millet increased gradually. *Roseiflexus*, a thermophilic, filamentous photosynthetic bacterium lacking chloroplasts, can grow under both light and dark conditions [75] and is a potentially beneficial microorganism [76]. Bacteria of the genus *Pseudarthrobacter* can protect plants from various biotic and abiotic stresses. The strain *Pseudarthrobacter* NIBRBAC000502770 promotes plant growth and increases flavonoid content [77], thereby inducing plants to secrete more flavonoid glycosides into the rhizosphere. These glycosides are then broken down into aglycones, which play antioxidant and metal-chelating roles in nutrient acquisition [78,79]. This process indirectly regulates the symbiotic relationship between broomcorn millet and microorganisms, thereby promoting plant growth. *Streptomyces* bacteria produce volatile organic compounds (VOCs), which can not only promote their own growth but also enhance the communication between related species in a dense microbial community to improve the common mechanism of microbial inbreeding [80], and the volatile organic compounds (VOCs) released by plants also affect the plant’s microbial community [81]. The long-distance diffusion of volatile organic compounds [propionaldehyde, γ-nonlactone, and dimethyl disulfide] in roots can attract bacteria with antifungal properties [82]. The abundance of the two in the rhizosphere of Yanshu No.10, with weak drought tolerance, showed a gradual downward trend, which may be related to the broomcorn millet variety [29]. Ren‘s study showed that the internal physiological and biochemical mechanisms of different varieties of broomcorn millet were not the same when resisting drought stress [16]. This may lead to the types of rhizosphere secretions of different genotypes of broomcorn millet, thus affecting the rhizosphere environment and microbial diversity. After rehydration, root physiological indexes, such as SOD activity, POD activity, MDA content, and osmotic adjustment substance proline content, were restored to varying degrees [83]. Therefore, we speculate that microorganisms begin to accumulate under the influence of plant growth status or the external environment [84] to maintain soil fertility and structure to assist plants in stress repair [85], and microbial groups continue to change over time [86]. In order to further explore the physiological mechanisms of the interaction between broomcorn millet and its rhizosphere microorganisms after rehydration, we need to use better experimental methods in order to determine the material circulation between plants, microorganisms, and their environments under natural conditions.

## 5. Conclusions

In this study, two kinds of broomcorn millet with different levels of drought resistance were used as the study materials. High-throughput sequencing technology was used to explore the effects of rewatering after drought stress during the flowering stage on bacterial diversity and community structure in the rhizosphere of broomcorn millet. This study has shown that 10 days and 20 days of rehydration have different effects on the abundance and diversity of bacteria in the rhizosphere of broomcorn millet. There were differences in the abundance and variation of rhizosphere bacteria during the rehydration process of broomcorn millet. In the rhizosphere microorganisms of different genotypes of broomcorn millet, the abundance of different bacteria varied. These results may help us to understand the state of broomcorn millet and its rhizosphere microorganisms after seasonal drought.

## Figures and Tables

**Table 1:** The results of three-way analysis of variance (*n* = 3) for the effects of cultivar variety, treatment, and sampling period after rewatering on the richness and evenness of bacterial community in the rhizosphere of broomcorn millet.

Factor	OTU		Shannon Index	
	F	*p*	F	*p*
Cultivar	1.404	0.253	1.887	0.188
Treatment	1.009	0.330	2.791	0.114
Period	10.041	<0.01	0.233	0.636
Cultivar × Treatment	1.606	0.223	3.045	0.100
Cultivar × Period	0.001	0.982	0.112	0.743
Treatment × Period	0.168	0.687	0.647	0.433
Cultivar × Treatment × Period	0.340	0.568	0.430	0.521

## Data Availability

The original contributions presented in the study are included in the article, further inquiries can be directed to the corresponding authors.
